# Examining the Association between Mitochondrial Genome Variation and Coronary Artery Disease

**DOI:** 10.3390/genes13030516

**Published:** 2022-03-15

**Authors:** Baiba Vilne, Aniket Sawant, Irina Rudaka

**Affiliations:** 1 Bioinformatics Lab, Rīga Stradiņš University, LV-1007 Riga, Latvia; aniket.sawant@rsu.lv; 2 Scientific Laboratory of Molecular Genetics, Rīga Stradiņš University, LV-1007 Riga, Latvia; irina.rudaka@rsu.lv

**Keywords:** coronary artery disease, mitochondria, mitochondrial DNA variants, haplogroups, association, common and rare variants

## Abstract

Large-scale genome-wide association studies have identified hundreds of single-nucleotide variants (SNVs) significantly associated with coronary artery disease (CAD). However, collectively, these explain <20% of the heritability. Hypothesis: Here, we hypothesize that mitochondrial (MT)-SNVs might present one potential source of this “missing heritability”. Methods: We analyzed 265 MT-SNVs in ~500,000 UK Biobank individuals, exploring two different CAD definitions: a more stringent (myocardial infarction and/or revascularization; HARD = 20,405), and a more inclusive (angina and chronic ischemic heart disease; SOFT = 34,782). Results: In HARD cases, the most significant (*p* < 0.05) associations were for m.295C>T (control region) and m.12612A>G (ND5), found more frequently in cases (OR = 1.05), potentially related to reduced cardiorespiratory fitness in response to exercise, as well as for m.12372G>A (ND5) and m.11467A>G (ND4), present more frequently in controls (OR = 0.97), previously associated with lower ROS production rate. In SOFT cases, four MT-SNVs survived multiple testing corrections (at FDR < 5%), all potentially conferring increased CAD risk. Of those, m.11251A>G (ND4) and m.15452C>A (CYB) have previously shown significant associations with body height. In line with this, we observed that CAD cases were slightly less physically active, and their average body height was ~2.00 cm lower compared to controls; both traits are known to be related to increased CAD risk. Gene-based tests identified CO2 associated with HARD/SOFT CAD, whereas ND3 and CYB associated with SOFT cases (*p* < 0.05), dysfunction of which has been related to MT oxidative stress, obesity/T2D (CO2), BMI (ND3), and angina/exercise intolerance (CYB). Finally, we observed that macro-haplogroup I was significantly (*p* < 0.05) more frequent in HARD cases vs. controls (3.35% vs. 3.08%), potentially associated with response to exercise. Conclusions: We found only spurious associations between MT genome variation and HARD/SOFT CAD and conclude that more MT-SNV data in even larger study cohorts may be needed to conclusively determine the role of MT DNA in CAD.

## 1. Introduction

Coronary artery disease (CAD) and its major complication myocardial infarction is the most common cardiovascular disease and the main leading cause of morbidity and mortality worldwide. CAD is posing a huge socio-economic burden to society and health systems [1] and its prevalence is expected to increase in the coming years [2,3,4]. CAD is a multifactorial disease with complex etiology, considered to be driven by both environment/lifestyle and genetic factors [5,6,7]. Over the last 14 years, several large-scale genome-wide association studies and their meta-analysis have identified numerous common genetic variants associated with CAD risk [8,9,10,11,12,13,14,15,16,17] and explored their functional consequences [18,19,20,21,22,23,24,25,26,27]. However, collectively, these variants explain only a small proportion (~20%) of the disease heritability [12,28]. Genetic variations of the mitochondrial (MT) DNA have remained out of focus for a long time and present an underexplored potential source of the “missing heritability” of several complex traits, including CAD [29,30,31].

The human MT DNA is a maternally-inherited, double-stranded, circular, histone-free “chromosome” of 16,596 base pairs (bp). Each mitochondrion contains 2 to 10 copies of MT DNA and, depending on the tissue energy requirement, each human cell may contain hundreds of mitochondria [32]. MT DNA encodes 37 genes corresponding to subunits ND1 to 6 (and 4 L) of the respiratory complex I, catalytic subunits I–III (CO1–3) of the cytochrome c oxidase (respiratory complex IV), subunits adenosine triphosphate 6 and 8 (ATP6 and 8) of the F1F0 ATPase and cytochrome b of the respiratory complex III. The remaining genes encode 2 ribosomal RNAs (16 S and 12 S rRNAs) and 22 transfer RNAs (tRNAs), used for mitochondrial protein synthesis [31,33,34]. All of them are involved in oxidative phosphorylation (OXPHOS), the process by which ATP, the major source of energy, is synthesized [35,36,37].

A toxic by-product of OXPHOS is the production of reactive oxygen species (ROS), unstable compounds which can generate free radicals [38]. Mitochondria are the primary source of endogenous ROS [38]. By antioxidant defense, cells can manage a certain level of free radical production. However, if threshold levels are exceeded, a state of oxidative stress occurs [39], which is known to play a vital role in the pathogenesis of atherosclerosis and CAD [24,40]. Many of the common CAD risk factors such as age, hypertension, hyperglycemia, high cholesterol levels, reduced physical activity, and smoking are also known to perturb mitochondrial function and increase oxidative stress [31].

The role of mitochondrial dysfunction in CAD etiology is well established, nevertheless, the role of the mitochondrial genome (DNA) in this process has not been extensively investigated [31]. Although several forms of cardiovascular disease have been related to the presence of pathogenic mitochondrial genome mutations, the vast majority of mitochondrial genetic variations are “natural” single-nucleotide variants (SNVs), not directly linked to disease pathogenesis [31]. During evolution, a number of such MT-SNVs have accumulated in mitochondrial genomes subdividing the human population into several discrete (geographic region-specific) mitochondrial phylogenetic clades or haplogroups [41]. As the mitochondrial genome does not undergo DNA recombination, haplogroups are relatively stable and enable the clustering of individuals based on their shared maternal ancestry [41]. These clusters are often associated with different racial/ethnic groups [31]. Considering that family history and race/ethnicity are known to influence CAD risk, it is reasonable to assume that mitochondrial haplogroups may contribute to this heritable modulator of CAD susceptibility [31].

**Figure 1:**
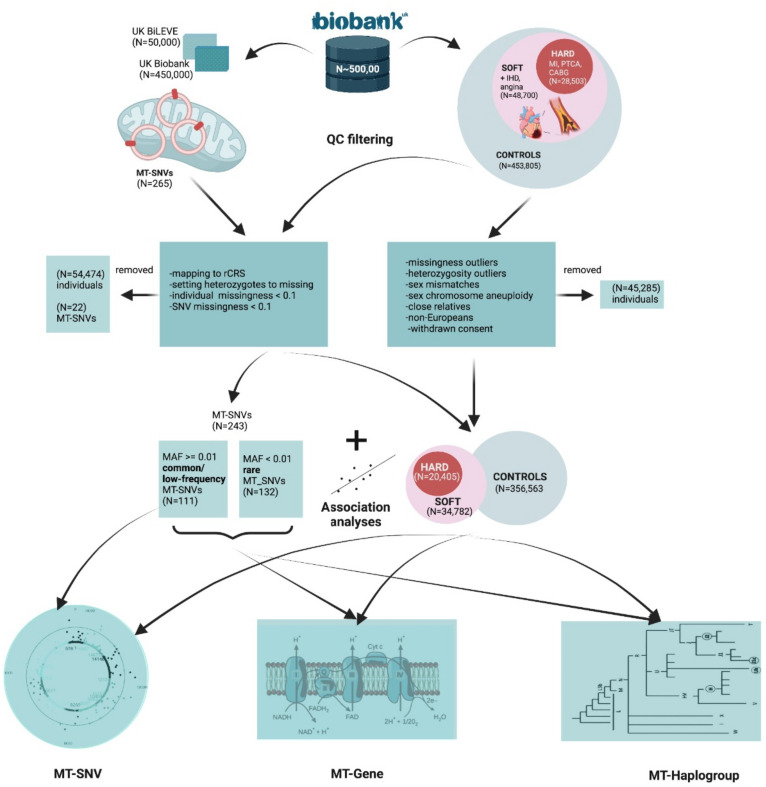
A complete workflow of the analyses performed. Created with Biorender.com (accessed on 22 February 2022).

In this study, we hypothesize that mitochondrial genome variation might present one potential source of the so-called “missing heritability” of CAD. To explore this hypothesis, we performed: (1) association analyses of common/low-frequency MT-SNVs (MAF > 0.01; *n* = 111) with CAD; (2) gene-based tests to investigate the cumulative impact of all MT-SNVs on the mitochondrial genes in relation to CAD; and (3) comparisons of mitochondrial haplogroup frequencies of individuals with CAD. In all cases, we explored two different CAD definitions (as previously used by [15]): a more stringent (HARD = 20,405), considering only myocardial infarction and/or revascularization, and a more inclusive (SOFT = 34,782), including all HARD CAD cases, as well as angina and chronic ischemic heart disease vs. controls in a cohort of ca. 500,000 UK Biobank individuals. The complete workflow of this analysis is summarized in Figure 1.

## 2. Materials and Methods

### 2.1. Study Population, Disease Phenotypes and Quality Filtering

The UK Biobank [42] is a large population-based prospective cohort study from the United Kingdom with genetic and deep phenotypic (~7221 phenotypes http://www.nealelab.is/uk-biobank, accessed on 22 February 2022) data on ca. 500,000 individuals aged 40 to 69. We downloaded these data (application ID 61684) and used a similar CAD case definition, as previously described by [15], for UK Biobank. HARD CAD cases included individuals with fatal or non-fatal myocardial infarction (MI), percutaneous transluminal coronary angioplasty (PTCA), or coronary artery bypass grafting (CABG). SOFT CAD included individuals meeting the HARD CAD definition as well as those with chronic ischemic heart disease (IHD) and angina (Figure 1). In HESIN hospital episodes data and death registry data from diagnosis and operation (primary and secondary causes), MI was defined as hospital admission or cause of death due to ICD9 410-412, ICD10 I21-I24, I25.2; PTCA was defined as hospital admission for PTCA (OPCS-4 K49, K50.1, K75); CABG was defined as hospital admission for CABG (OPCS-4 K40-K46); and angina or chronic IHD was defined as hospital admission or death due to ICD9 413, 414.0, 414.8, 414.9, ICD10 I20, I25.1, I25.5-I25.9. In UK Biobank self-reported data, cases were defined as having “vascular/heart problems diagnosed by the doctor” or “non-cancer illnesses that self-reported as angina or heart attack”. Self-reported surgery included PTCA, CABG, or triple heart bypass. All participants not defined as CAD cases using the SOFT definition were considered as controls in the analysis. For a complete list of definition codes, see Supplementary Table S1. We subsequently performed individual-level filtering (Figure 1) by removing missingness or heterozygosity outliers, participants with self-reported vs. genetically inferred sex mismatches or putative sex chromosome aneuploidy, individuals that were not of European (EUR) ancestry, and individuals having withdrawn their consent at the time of analysis. We also identified closely related participants (kinship coefficient > 0.088 i.e., first- or second-degree relative pairs), preferentially retaining CAD cases or relatives with the highest call rate.

The following individual characteristics were also extracted in order to characterize HARD/SOFT CAD cases vs. controls: age at recruitment (field #21022), sex (field #31), BMI (field #21001), height (field #50), hypertension (fields #4080 and #4079), hypercholesterolemia (self-reported data and ICD9/10) and (self-reported) use of cholesterol-lowering drugs, insulin and blood pressure medications (field #6153), type 2 diabetes (T2D, fields #41201, #41202 and #4120, E11), glycemic control, obesity, smoking status (“ever smoked”: field #20160 and “current” from “smoking status” field #20116), family history of heart disease (fields #20107, #20110, #20111 in a 1st-degree relative, i.e., father, mother or sibling, respectively). Data at the time of first assessment were obtained and processed to binary (yes/no) values or mean values for fields with continuous data with multiple readings at the time of first assessment.

### 2.2. Genotype Data Quality Control

In the UK Biobank [42], genotyping was performed using Affymetrix UK biobank Axiom (450,000 samples) and Affymetrix UK BiLEVE Axiom (50,000 samples) arrays (Figure 1) and the autosomal genetic data were subsequently imputed to the Haplotype Reference Consortium panel and UK10K4 + 1000 Genomes panel. We downloaded the genotype data for the 265 MT DNA variants for all 500,000 individuals and pre-processed MT DNA data as previously described in ref. [43]. In brief, we first made sure that the reference alleles match the latest MT Cambridge Revised Sequence (rCRS) of the Human MT DNA positions. After setting all potential heterozygotes to missing, further quality control of genotyped individuals included filtering for missingness by individual < 0.1 and missingness by SNV < 0.1 with PLINK [44]. For common/low-frequency variant association analyses, we also required that the minor allele frequency (MAF) > 0.01. An overview of the filtering of MT-SNVs is provided in Figure 1.

### 2.3. MT-SNV Association Analyses

For common and low-frequency (MAF > 0.01; *n* = 111) variants, we performed single marker tests to explore their associations with HARD and SOFT CAD (Figure 1) using SNPTEST v2.5.4 with the frequentist test and expected method, as previously described by ref. [45]. We used as covariates the array (UK Biobank vs. UK BiLEVE), sex, birth year, and the first five principal components of the autosomal genotype data, provided by the UK Biobank, similar to ref. [15] and Benjamini–Hochberg (BH) [46] adjustment for multiple testing was applied to calculate the false discovery rate (FDR). MT-SNV annotations were performed using a manually-curated database, HmtVar (https://www.hmtvar.uniba.it/, accessed on 22 February 2022).

### 2.4. MT-Gene-Based Association Analyses

To also consider the potential effects of rare (MAF ≤ 0.01) variants on CAD risk, we assigned all SNVs to MT genes based on MITOMAP (https://www.mitomap.org/MITOMAP, accessed on 22 February 2022) and used the R software package SKAT (v2.0.1) [47] to perform MT-gene-based (additionally including the whole mitochondrion as our region of interest, MT) association analyses with HARD and SOFT CAD phenotypes (Figure 1), again using as covariates the array (UK Biobank vs. UK BiLEVE), sex, birth year and the first five principal components of the autosomal genotype data, provided by the UK Biobank, similar to ref. [15] and obtain resampled residuals (*n*.Resampling = 1000, type.Resampling = “bootstrap”) to compute resampling *p*-value.

### 2.5. Haplogroup Assignment

We used the PhyloTree Build 17 [48] as implemented in HaploGrep (v2.2.8) [49] to estimate mitochondrial haplogroups in our dataset. Thereafter, we assigned individuals to one of the major European haplogroups (H, I, J, K, R, T, U, V, W, X), or to a group of “others” (https://www.mitomap.org/foswiki/pub/MITOMAP/WebHome/simple-tree-mitomap-2019.pdf, accessed on 22 February 2022). Fisher’s exact test [50] was used to calculate the statistical significance of the overlaps between haplogroups and HARD and SOFT CAD phenotypes. Benjamini–Hochberg (BH) [46] adjustment for multiple testing was applied to calculate the false discovery rate (FDR) (Figure 1).

## 3. Results

### 3.1. Characteristics of Study Subjects

The current study included ca. 500,000 genotyped individuals from the UK Biobank [42], 48,700 with an inclusive CAD phenotype (SOFT) that incorporated self-reported angina or other evidence of chronic coronary heart disease, of which 28,503 had a more stringently defined CAD phenotype (HARD) of myocardial infarction (Figure 1), similar to ref. [15]. All participants (*n* = 453,805) not defined as CAD cases using the SOFT definition are considered as controls in the analysis. After this step of quality control, 45,285 individuals were removed: 24,770 cases with the HARD, 42,079 cases with the SOFT CAD phenotype, and 415,271 controls remained. Finally, further quality control of genotyped individuals included filtering for missingness by individual < 0.1 and missingness by SNV < 0.1 was performed, considering the 265 mitochondrial variants present on the UK Biobank or UK BiLEVE arrays (as described in Section 2.2. in Methods). As a result, a further 54,474 individuals were removed, leaving us with 20,405 cases with the HARD and 34,782 cases with the SOFT phenotype vs. 356,563 controls. Individual characteristics of these individuals, in terms of common CAD risk factors, are summarized in Table 1.

### 3.2. MT-SNV Associations with HARD and SOFT CAD Phenotypes

After quality control of genotyped individuals (including filtering for missingness by individual < 0.1 and missingness by SNV < 0.1, as described in Section 2.2. in Methods), from the 265 MT-SNVs present in the UK Biobank or UK BiLEVE arrays, 243 remained for further analyses. For the genotyped common and low-frequency MT-SNVs (MAF > 0.01; *n* = 111, of those *n* = 39 with MAF > 0.05; genotyping rate > 0.99) in the UK Biobank, we performed single marker association analyses with HARD (*n* = 20,405) and SOFT (*n* = 34,782) CAD phenotypes, adjusting for the array, sex, birth year and first five principal components.

**Figure 2:**
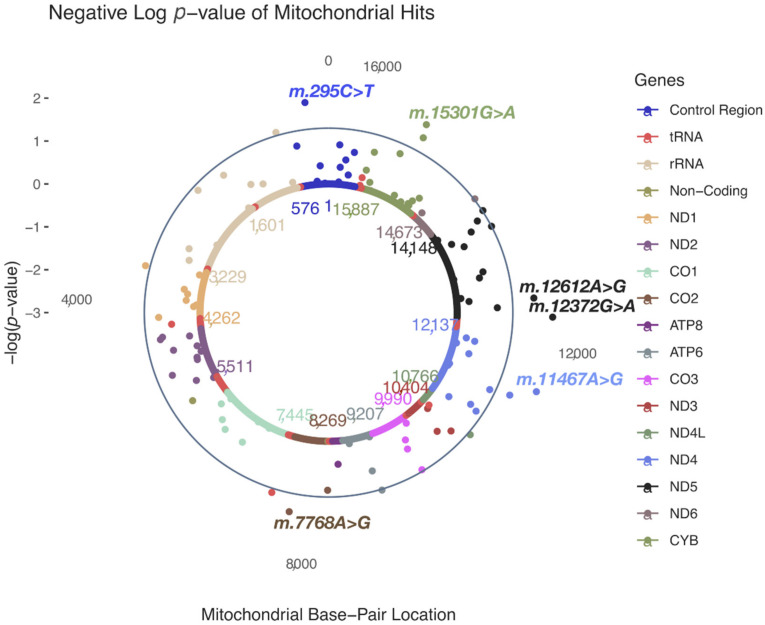
A solar plot of HARD CAD common and low-frequency (MAF > 0.01; *n* = 111) MT-SNV associations.

In HARD cases, no MT-SNVs survived multiple testing correction, the most significant (nominal *p* < 0.05) findings (Table 2 and Figure 2) being for m.295C>T (rs41528348, *p* = 0.0118, MAF = 0.10, OR = 1.05; 95% CI 1.02–1.09, in control region/CR, tagging macro-haplogroup J) and m.12612A>G (rs28359172, *p* = 0.0158, MAF = 0.10, OR = 1.05; 95% CI 1.02–1.08, synonymous, in ND5 gene, tagging macro-haplogroup J), both more frequent in cases, thus potentially conferring increased CAD risk. In addition, four more MT-SNVs were found more frequently in controls: m.12372G>A (rs2853499, *p* = 0.0059, MAF = 0.22, OR = 0.97; 95% CI 0.95–0.99, synonymous, in ND5 gene, tagging macro-haplogroup U), m.11467A>G (rs2853493, *p* = 0.0065, MAF = 0.22, OR = 0.97; 95% CI 0.95–1.00, synonymous, in ND4 gene, tagging macro-haplogroup U), m.15301G>A (rs193302991, *p* = 0.0115, MAF = 0.04, OR = 0.97; 95% CI 0.92–1.03, synonymous, in CYB gene) and m.7768A>G (rs41534044, *p* = 0.0185, MAF = 0.04, OR = 0.91; 95% CI 0.86–0.96, synonymous, in CO2 gene). For a complete list of MT-SNV associations with HARD CAD phenotypes, see Supplementary Table S2.

**Figure 3:**
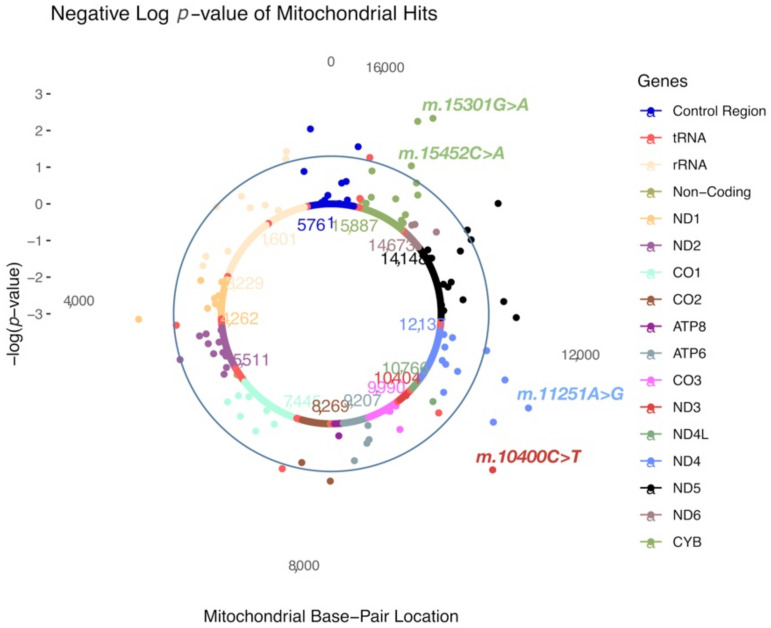
A solar plot of SOFT CAD common and low-frequency (MAF > 0.01; *n* = 111) MT-SNV associations. CR = control region.

In SOFT cases, four MT-SNVs survived multiple testing correction (at FDR < 5%; Table 3 and Figure 3), all potentially conferring increased CAD risk: m.10400C>T (rs28358278, *p* = 0.0007, MAF = 0.02, OR = 1.28; 95% CI 1.21–1.35, non-synonymous/Thr→Ala, in ND3 gene, tagging macro-haplogroup M), m.11251A>G (rs869096886, *p* = 0.0011, MAF = 0.20, OR = 1.03; 95% CI 1.01–1.05, synonymous, in ND4 gene, tagging macro-haplogroups J and T), and two MT-SNVs in CYB gene—m.15452C > A (rs193302994, *p* = 0.0017, MAF = 0.20, OR = 1.03; 95% CI 1.01–1.05, non-synonymous/Leu→Ile, tagging macro-haplogroups J and T) and m.15301G>A (rs193302991, *p* = 0.0010, MAF = 0.04, OR = 1.03; 95% CI 0.99–1.07, synonymous). For a complete list of MT-SNVs associations with SOFT CAD phenotype, see Supplementary Table S3.

### 3.3. MT-Gene-Based Associations with HARD and SOFT CAD Phenotypes

We next sought to also consider the potential effects of rare (MAF < 0.01) MT-SNVs on CAD risk, hence we performed MT-gene-based association analyses with HARD and SOFT CAD phenotypes, additionally including the whole mitochondrion as our region of interest (MT). As a result, we observed that in both HARD and SOFT cases, CO2 displayed gene-based association at nominal significance (*p* < 0.05), while CYB and ND3 were also associated (nominal *p* < 0.05) with SOFT CAD phenotype (Table 4). When considering the whole mitochondrion (MT), no significant associations with CAD were observed (*n* = 243; *p* = 0.07, Table 4).

### 3.4. MT-Haplogroup Associations with HARD and SOFT CAD Phenotypes

Different human mitochondrial haplogroups may result in differences in mitochondrial function that may influence susceptibility to CAD. Hence, we estimated all the mitochondrial haplogroups in our dataset (Table 5, Supplementary Tables S4 and S5).

Three haplogroups survived multiple testing correction (at FDR < 5%) in both HARD and SOFT cases vs. controls: M45a (0.59% and 0.55% vs. 0.39%, OR = 1.52 and OR = 1.42, respectively), G2b1a2 (0.28% and 0.26% vs. 0.16%, OR = 1.73 and OR = 1.60, respectively) and U2b2 (0.11% and 0.09% vs. 0.04%, OR = 2.56 and OR = 2.31, respectively). In HARD cases, haplogroup M57b1 was also significantly (at FDR < 5%) over-represented in cases vs. controls (0.02% vs. <0.01%, OR = 3 3.06), while haplogroup L2c was significantly (at FDR < 5%) under-represented in cases vs. controls (0.01% vs. 0.07%, OR = 0.18) (Table 5 and Supplementary Table S4). In SOFT cases, haplogroup M3a was also significantly (at FDR < 5%) over-represented in cases vs. controls (0.21% vs. 0.13%, OR = 1.67, Table 5 and Supplementary Table S5).

**Figure 4:**
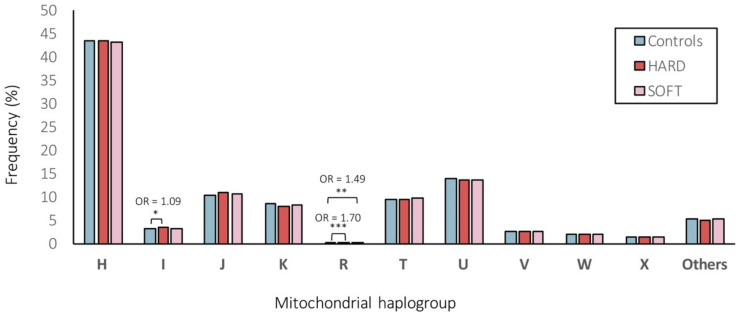
Frequencies (%) of mitochondrial (MT) haplogroups within HARD and SOFT CAD phenotypes vs. controls. ***, ** and * represent statistically significant (of *p* < 0.001, *p* < 0.01 and *p* < 0.05, respectively) differences between the comparison groups.

We further assigned individuals to one of the major European haplogroups (Figure 4). As a result, 43.28%, 3.19%, 10.70%, 8.25%, 0.22%, 9.52%, 13.70%, 2.65%, 2.01%, 1.34% and 5.14% of individuals belonged do the haplogroups H, I, J, K, R, T, U, V, W, X or “others”, respectively. Overall, the frequencies of the major European mitochondrial haplogroups did not differ significantly (at FDR < 5%) between CAD patients and control subjects (Figure 4). Only the frequency of haplogroup I was significantly (nominal *p* < 0.05) higher in patients with HARD CAD phenotype vs. controls (3.35% vs. 3.08%, OR = 1.09) and the haplogroup R was significantly (nominal *p* < 0.001 and *p* < 0.01) higher in patients with HARD and SOFT CAD phenotype vs. controls (0.26% and 0.23% vs. 0.16%, OR = 1.70 and OR = 1.49, respectively; Figure 4).

## 4. Discussion

Over the last 14 years, several large-scale genome-wide association studies have found hundreds of single-nucleotide variants (SNVs) significantly associated with CAD; however, these explain <20% of the heritability. In this study, we hypothesize that mitochondrial (MT)-SNVs might present one potential source of the “missing heritability”.

We analyzed 265 common/low-frequency (MAF ≥ 1%) and rare (MAF < 1%) MT-SNVs in ~500,000 UK Biobank individuals, exploring two different CAD definitions, HARD (*n* = 20,405) and SOFT (*n* = 34,782) (Figure 1), as previously proposed by [15], and using the array, sex, birth year and first five principal components as covariates. Overall, the differences in the prevalence of common risk factors among CAD cases (both HARD and SOFT phenotypes) and controls were statistically significant (*p* < 0.001; Table 1), male gender, older age, hypertension, hypercholesterolemia, obesity, T2D, physical inactivity, shorter body statue, smoking and positive family history demonstrating predominance in CAD patients.

**Figure 5:**
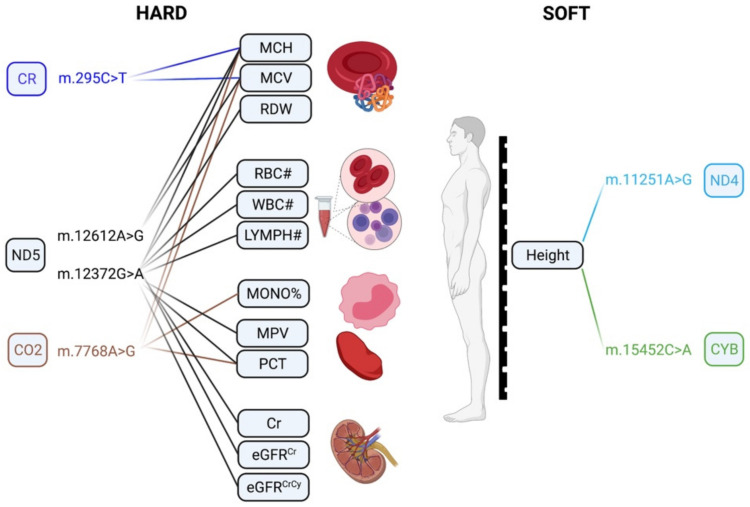
A visual overview of the previous findings for HARD and SOFT CAD MT-SNV associations in UK Biobank reported by ref. [58]. Created with BioRender.com, accessed on 22 February 2022. MCH: mean corpuscular hemoglobin; MCV: mean corpuscular volume; RDW: red blood cell distribution width; RBC#: red blood cell count; WBC#: white blood cell count; LYMPH#: lymphocyte count; MONO%: monocyte percentage of white cells; MCV: mean corpuscular volume; PCT: plateletcrit; Cr: creatinine; eGFRCr: estimated glomerular filtration rate creatinine; eGFRCrCy: estimated glomerular filtration rate creatinine and cystatin C.

When performing common and low-frequency MT-SNVs (MAF ≥ 0.01; *n* = 111) association analyses in these individuals, we observed that in HARD cases, no MT-SNVs survived multiple testing correction, the most significant (nominal *p* < 0.05) findings being for m.295C>T, m.12612A>G, m.12372G>A, m.11467A>G, m.15301G>A and m.7768A>G (Table 2 and Figure 2). m.295C>T (rs41528348, *p* = 0.0118, MAF = 0.10) is a control region (CR) genetic variant tagging macro-haplogroup J, known to be associated with low maximal oxygen uptake (VO2max) in response to aerobic exercise [51,52,53] and thus cardiorespiratory fitness and CVD risk [54,55,56,57]. In line with this, a previous study in the UK Biobank [58] reported a significant association between m.295C>T and several blood cell traits (Figure 5), known to increase with training [59]. Our results demonstrate that m.295C>T was more frequent (OR = 1.05; 95% CI 1.02–1.09, *p* = 0.0118) in HARD cases, thus potentially conferring a decreased cardiorespiratory fitness/exercise capacity and increased CAD risk. m.12612A>G (rs28359172, *p* = 0.0158, MAF = 0.10), a synonymous (V92V) genetic variant in the subunit 5 of NADH dehydrogenase (ND5), may demonstrate similar functionality, as it is also tagging macro-haplogroup J and displays similar patterns of association in the UK Biobank (Figure 5) [58].

m.12372G>A (rs2853499, *p* = 0.0059, MAF = 0.22) and m.11467A>G (rs2853493, *p* = 0.0065, MAF = 0.22) represent two synonymous (L12L and L236L) variants in the ND5 and ND4 (the subunit 4 of NADH dehydrogenase) genes found more frequently in control vs. HARD CAD cases (OR = 0.97; 95% CI 0.95–0.99 and OR = 0.97; 95% CI 0.95–1.00), tagging macro-haplogroup U. This possibly CAD-protective (and longevity increasing [60]) role of macro-haplogroup U could be partially explained by altered pH [61] and a reduced load of harmful reactions [62], as pH is known to play a role in mitochondrial ROS generation [63,64] and endurance time during exercise [65]. Interestingly, m.12372G>A displayed significant associations with ten different blood cell and kidney-related traits in the UK Biobank (Figure 5) [58]. Endurance time during exercise has been related to pre-exercise blood pH and demonstrated to increase with increasing pH [65].

In SOFT cases, four MT-SNVs survived multiple testing correction (at FDR < 5%; Table 3 and Figure 3), all potentially conferring increased CAD risk: m.10400C>T, m.11251A>G, m.15452C>A and 15301G>A. m.11251A>G (rs869096886, *p* = 0.0011, MAF = 0.20) represents a synonymous sequence variant in the ND4 gene and m.15452C>A (rs193302994, *p* = 0.0017, MAF = 0.20) is a non-synonymous (Leu→Ile) sequence variant in the CYB gene; both were found more frequently in SOFT CAD cases vs. controls (OR = 1.03; 95% CI 1.01–1.05). m.11251A>G (rs869096886) and m.15452C>A (rs193302994) are tagging macro-haplogroup J and thus potentially related to a decreased cardiorespiratory fitness/exercise capacity [52,53] and increased CAD risk. Moreover, both MT-SNVs displayed significant associations with body height in the UK Biobank (Figure 5) [58]. In line with this, we observed that the average body height of both male and female CAD cases was ~2.00 cm lower compared to controls (Table 1), and shorter body height is related to an increased CAD risk [66].

Gene-based tests revealed that in both HARD and SOFT cases, CO2 displayed gene-based association at nominal significance (*p* < 0.05, Table 4). The CO2 gene encodes for the second subunit of cytochrome c oxidase (COX, complex IV). Dysfunction of COX has been previously associated with mitochondrial oxidative stress, obesity, and T2D [67]. CYB and ND3 were also associated (nominal *p* < 0.05) with the SOFT CAD phenotype (Table 4). Somatic variations in CYB have been previously related to hypertrophic cardiomyopathy (one of its clinical manifestations being angina) and exercise intolerance [68]. Recently, a large gene-based meta-analysis of mitochondrial genes with several metabolic traits identified ND3 associated with BMI (*p* < 1 × 10^−3^) [43].

All haplogroups demonstrating significant (at FDR < 5%) associations in our study (M45a, G2b1a2, U2b2 with both HARD/SOFT, M57b1 and L2c (under-represented) with HARD and M3a with SOFT CAD phenotypes; Table 5 and Supplementary Table S4) were with a frequency <1%, whereas other studies have considered only haplogroups with a frequency ≥5% [69]. Low counts in the less common haplogroups may lead to a false-positive result [70]. Although this should be addressed by performing multiple testing corrections, grouping the less frequent haplogroups may be another approach to tackle this [70]. Hence, we also assigned individuals to one of the major European haplogroups (Figure 4) for comparison. As a result, we observed that 43.28% of the individuals belonged to the macro-haplogroup H, 13.70% to the macro-haplogroup U, 10.70% to the macro-haplogroup J, and 9.52% to the macro-haplogroup T (Figure 4), in line with previous reports in other European populations [71]. Overall, the frequencies of the major European mitochondrial haplogroups did not differ significantly (at FDR < 5%) between CAD patients and control subjects (Figure 4). Only the frequency of haplogroup I was significantly (nominal *p* < 0.05) higher in patients with HARD CAD phenotype vs. controls (3.35% vs. 3.08%, OR = 1.09) and the macro-haplogroup R was significantly (nominal *p* < 0.001 and *p* < 0.01) higher in patients with HARD and SOFT CAD phenotype vs. controls (0.26% and 0.23% vs. 0.16%, OR = 1.70 and OR = 1.49, respectively) (Figure 4). Of note, however, we were able to assign most samples reliably into haplogroups, as the MT DNA haplogroups were deduced from genotyping arrays with limited numbers of (high-quality) SNVs being profiled, hence the quality score for haplogroup assignment ranged from 0.50 to 0.86, with a median of 0.68. Therefore, we were not able to exclude samples with quality scores for haplogroup assignment <0.8 (as in ref. [72]). Moreover, we performed Fisher’s exact, which did not allow us to adjust for covariates, hence it is possible that known and unknown potential confounding factors might have influenced these results. Though, in which cases to adjust for which covariates and whether it will increase or decrease the study power and/or bias, is still a matter of intense debate [73,74].

Several other limitations should also be acknowledged. It is well known that very large cohorts are required to reliably associate genetic variations with complex traits [70]. The power for detecting causal MT-SNVs and haplogroups has been compared with that in the nuclear genome given equal effect sizes, estimating that the sample size required for the mitochondrial studies would be roughly the same as that needed for the nuclear genome studies [75]. Previous power calculations for ischemic stroke (assuming an additive model) [76] revealed a maximum power of 73% to detect SNVs with OR = 1.4 and MAF = 0.30, whereas for SNVs conferring OR = 1.20 and MAF = 0.20, the study power dropped to 4.6% and further to 0.001% for OR = 1.10 and MAF = 0.10. This study concluded that “prohibitively large sample sizes” would be required to achieve sufficient power to detect individual MT DNA variants [76]. In line with this, we observe that in HARD CAD cases, where *n* = 20,405, no MT-SNVs survived multiple testing correction, whereas when increasing *n* to 34,782 in SOFT CAD cases, four MT-SNVs survived multiple testing correction (FDR < 5%). Hence, even larger sample sizes (≥50,000) may be required to reliably associate MT-SNVs and haplogroups with CAD.

In addition to the number of individuals, the number of MT-SNVs studied was also limited. In the UK Biobank [42], genotyping was performed using Affymetrix UK biobank Axiom (450,000 samples) and Affymetrix UK BiLEVE Axiom (50,000 samples) arrays, which included 265 genotyped MT DNA variants. After quality control procedures (Figure 1), 243 MT-SNVs remained for further analyses, and 111 of those were common or low-frequency (MAF > 0.01) and could be used for single-marker association analyses. However, this is clearly not a representative set of MT-SNVs and, as previously recognized, some regions may be not well covered, such as the hypervariable regions [77,78]. Clearly, whole-genome sequencing or targeted sequencing of MT-DNA, considering their ability to achieve a deep genome coverage, would allow the identification of many more MT-SNVs (especially the low-frequency/rare variants; MAF ≤ 0.01), improving also the detection of haplogroups and allowing the investigation of heteroplasmy, a phenomenon characteristic to MT DNA [33,79].

Heteroplasmy denotes the coexistence of MT DNA genomes with wild-type inherited SNVs and somatic variants in varying ratios, which are dynamically determined and display patterns of cell and tissue specificity, and may differ even within the same mitochondrion [33], determining the clinical presentation of disease phenotypes [77,80,81]. In this study, we were limited to genotype calls from arrays, which are restricted in terms of minor alleles and do not allow the capture of heteroplasmy [77,80]. Moreover, MT DNA content was assessed only in blood cells, whereas previous studies have identified an additional six vascular and metabolic tissues relevant to CAD [82,83]. Therefore, whole-genome sequencing/targeted sequencing of MT-DNA across several vascular and metabolic tissues relevant to CAD may be necessary [7,82,83] in order to characterize the full landscape of mitochondrial genetic variations and their potential contribution to these complex disease phenotypes. This may be necessary, especially considering that the energy requirements and thus sensitivity to the changes in mitochondrial function differ for different cells and tissues and hence may be important in determining the phenotypic effect of MT-SNVs [31].

We also did not consider mitochondrial DNA copy number (MT DNA-CN), representing the number of mitochondria per cell and the number of MT DNA per mitochondrion [84,85]. Each mitochondrion contains multiple copies of MT DNA, and different cells and tissues contain different numbers (up to 7000) of mitochondria, again displaying patterns of cell and tissue variability [84,85]. MT DNA-CN is believed to serve as an indirect biomarker that would capture the underlying mitochondrial activity and function, such as energy production capacity and metabolic characteristics, thus possibly playing a causative role in health and disease [85]. Decreased MT DNA-CN has been previously associated with an increased risk of developing cardiovascular disease (CVD) outcomes [84]. More recently, similar analyses in the UK Biobank demonstrated a possible causal role of lower MT DNA-CN on higher CAD risk [86]. In an even larger cohort (of 408,361 individuals from TOPMed and UK Biobank), a decline in MT DNA-CN was observed in elderly individuals (>65 years) and lower MT DNA-CN levels also demonstrated an age-independent association with hypertension, hyperlipidemia, T2D, and obesity, i.e., the well-known CAD risk factors [87]. However, none of these studies compared the MT DNA-CN levels between HARD vs. SOFT CAD phenotypes, which could be a subject of future studies. Furthermore, considering that MT DNA-CN varies greatly across cell and tissue types, again profiling of several vascular and metabolic tissues relevant to CAD may be necessary for such investigations [7,82,83].

Yet another important aspect not considered here is the nuclear genome, considering the co-evolution of mitochondria and eukaryotic cells [37]. The mitochondrial genome encodes only 37 genes, mainly components of the OXPHOS machinery, whereas the remaining ~1000–1500 mitochondrial proteins are all encoded by the nuclear genome [88]. The importance of common genetic variation in the nuclear genome regulating MT heteroplasmy and DNA-CN is an active area of research [85,89,90,91]. Moreover, genetic variants in nuclear genes could lead to oxidative disorders or modulate the mitochondrial variants [81], and mild nuclear gene variants could potentially become clinically relevant when combined with an incompatible MT DNA [92]. Additive interactions (epistasis) between mitochondrial variants in the MT-ND2 gene and nuclear variants in genes responsible for mitochondrial replication and transcription have been demonstrated to influence the BMI and obesity phenotype [93]. Similarly, our previous investigations have demonstrated the role of nuclear-encoded mitochondria imported genes in coordinating the response to hypercholesterolemia and atherosclerotic lesion expansion, as well as foam cell formation [24]. Hence, further analyses also considering these additional variations will be required.

Finally, similar to SNVs in the nuclear genome, even if (mitochondria) genome-wide significant associations with HARD/SOFT CAD phenotypes would be identified, their functional consequences would need to be determined in the CAD-relevant tissues [82,83]. Currently, functional studies for MT-SNVs are not readily available; however, several novel experimental animal models (e.g., mice strains displaying DNA haplogroups similar to those observed in humans) may be available in the near future, allowing the investigation of the potential causality of the relationship between inherited “natural” non-pathogenic MT-SNVs and potential alterations in mitochondrial function (e.g., oxygen consumption and oxidant production, cellular ATP levels) and their relation to alterations in cardiovascular function and CAD risk [31,37,81].

## 5. Conclusions

We found only spurious MT-SNV, gene, and haplogroup associations with HARD and SOFT CAD phenotypes and conclude that whole-genome sequencing/targeted-sequencing of MT-DNA, across several vascular and metabolic tissues relevant to CAD in even larger study cohorts (*n* > 50,000), followed by functional studies in animal models, may be necessary to conclusively determine the role of MT-SNVs, genes, and haplogroups in modulating the risk of CAD. Therefore, whole-genome sequencing/targeted sequencing of MT-DNA across several vascular and metabolic tissues relevant to CAD may be necessary [7,82,83] in order to characterize the full landscape of mitochondrial genetic variations and their potential contribution to these complex disease phenotypes. This may be especially necessary considering that the energy requirements and thus sensitivity to the changes in mitochondrial function differ for different cells and tissues and hence may be important in determining the phenotypic effect of MT-SNVs [31].

## Figures and Tables

**Table 1:** Individual characteristics of HARD and SOFT CAD cases vs. controls. *** Represents statistically significant (of *p* < 0.001) difference between HARD and SOFT CAD cases vs. controls, whereas +++, ++ and + represent statistically significant (of *p* < 0.001, *p* < 0.01 and *p* < 0.05, respectively) difference between HARD vs. SOFT CAD cases.

Risk Factors	HARD ***	SOFT ***	CONTROL
Men (%)	77	67	43
Age, years (mean ± SD, range)	63 (61.33 ± 6.35, 58–66)	63 (61.14 ± 6.43, 58–66)	57 (56.08 ± 8.05, 50–63)
Diastolic blood pressure > 90 mmHg (%)	21.23	21.54	22.41
Systolic blood pressure > 140 mmHg (%)	49.88	50.04	41.52
Hypercholesterolemia (%)	51.20	44.78	6.00
Hypertriglyceridemia (%)	1.55	1.43	0.80
Poor glycemic control (%)	3.59	3.20	0.80
Type 2 diabetes (%)	20.80	19.41	4.14
BMI, kg/m^2^ (mean ± SD, range)	28.29 (28.91 ± 4.72, 25.72–31.47)	28.34 (29.03 ± 4.98, 25.66–31.66)	26.51 (27.18 ± 4.69, 23.96–29.60)
Obesity (BMI > 30 kg/m^2^, %)	35.17	36.10	22.65
Central obesity (%)	63.39 ^+++^	60.40	36.61
Body height male (mean ± SD, range): female	173.92 ± 6.76 ^+++^ (Med = 174)	174.16 ± 6.82 (Med = 174)	175.95 ± 6.82 (Med = 176)
	160.37 ± 6.38 ^++^ (Med = 160)	160.68 ± 6.33 (Med = 161)	162.61 ± 6.29 (Med = 163)
Physically active (%)	51.31	51.08	54.60
Smoking history (ever smoked, %)	72.23 ^+++^	69.56	60.21
Current smoker (%)	13.74 ^+^	12.90	9.27
History of heart disease in first-degree relative (%)	59.14 ^+++^	57.35	41.41

**Table 2:** HARD CAD common and low-frequency (MAF > 0.01; *n* = 111) MT-SNV most significant associations. CR = control region.

Locus	RSID	Variation	MAF	AA	OR	95% CI	*p*	HG
*ND5*	rs2853499	m.12372G>A	0.22	Syn	0.97	0.95–0.99	0.0059	U
*ND4*	rs2853493	m.11467A>G	0.22	Syn	0.97	0.95–1.00	0.0065	U
*CYB*	rs193302991	m.15301G>A	0.04	Syn	0.97	0.92–1.03	0.0115	.
*CR*	rs41528348	m.295C>T	0.10	.	1.05	1.02–1.09	0.0118	J
*ND5*	rs28359172	m.12612A>G	0.10	Syn	1.05	1.02–1.08	0.0158	J
*CO2*	rs41534044	m.7768A>G	0.04	Syn	0.91	0.86–0.96	0.0185	.
*ND4*	rs28358285	m.11299T>C	0.08	Syn	0.94	0.91–0.98	0.0227	K
*CYB*	rs41518645	m.15257G>A	0.02	Asp→Asn	1.11	1.04–1.19	0.0231	.
*ND1*	rs28358584	m.3480A>G	0.08	Syn	0.94	0.91–0.98	0.0390	.
*tRNA^Ser(UCN)^*	rs201950015	m.7476C>T	0.02	.	1.01	1.03–1.19	0.0400	.
*rRNA^12S^*	rs2853518	m.750A>G	0.02	.	0.87	0.80–0.95	0.0420	.
*ND4L*	rs28358280	m.10550A>G	0.08	Syn	0.95	0.91–0.98	0.0435	K
*ND6*	rs193302977	m.14167C>T	0.08	Syn	0.95	0.91–0.98	0.0476	.
*ATP6*	rs193303045	m.9055G>A	0.09	Ala→Thr	0.95	0.92–0.98	0.0476	.
*ND5*	rs869156190	m.13965T>C	0.01	Syn	1.10	1.00–1.21	0.0484	.
*ND5*	rs28359178	m.13708G>A	0.12	Ala→Thr	1.04	1.01–1.07	0.0499	J

**Table 3:** SOFT CAD common and low-frequency (MAF > 0.01; *n* = 111) MT-SNV most significant associations. CR = control region. * MT-SNVs that survived multiple testing correction (at FDR < 5%).

Locus	RSID	Variation	MAF	AA	OR	95% CI	*p*	HG
*ND3*	rs28358278	m.10400C>T	0.02	Thr→Ala	1.28	1.21–1.35	0.0007 *	M
*CYB*	rs193302991	m.15301G>A	0.04	Syn	1.03	0.99–1.07	0.0010 *	.
*ND4*	rs869096886	m.11251A>G	0.20	Syn	1.03	1.01–1.05	0.0011 *	JT
*CYB*	rs193302994	m.15452C>A	0.20	Leu→Ile	1.03	1.01–1.05	0.0017 *	JT
*ND5*	rs869156190	m.13965T>C	0.01	Syn	1.13	1.05–1.21	0.0035	.
*ND4*	rs2857284	m.10873T>C	0.03	Syn	1.03	0.99–1.07	0.0048	.
*ND1*	rs1599988	m.4216T>C	0.20	Tyr→His	1.03	1.01–1.05	0.0055	.
*CR*	rs41528348	m.295C>T	0.10	.	1.04	1.01–1.06	0.0084	J
*ND4*	rs2853493	m.11467A>G	0.22	Syn	0.98	0.96–0.99	0.0084	U
*ND5*	rs2853499	m.12372G>A	0.22	Syn	0.98	0.96–0.99	0.0089	U
*ND5*	rs28359172	m.12612A>G	0.10	Syn	1.03	1.01–1.06	0.0190	J
*CR*	rs41419246	m.16145G>A	0.03	.	1.08	1.03–1.12	0.0242	.
*CYB*	rs41518645	m.15257G>A	0.02	Asp→Asn	1.08	1.02–1.14	0.0256	.
*rRNA^12S^*	rs2853517	m.709G>A	0.15	.	1.04	1.01–1.01	0.0256	L6, G, N2, T, B5
*CO2*	.	m.8269G>A	0.03	Syn	1.06	1.02–1.11	0.0273	.
*tRNA^Ser(UCN)^*	rs201950015	m.7476C>T	0.02	.	1.08	1.02–1.14	0.0371	.
*rRNA^12S^*	rs2853518	m.750A>G	0.02	.	0.90	0.84–0.96	0.0392	.

**Table 4:** MT-gene-based associations with HARD and SOFT CAD phenotypes. MT = mitochondrion; *n* (ALL) = the number of MT-SNVs in the region; *n* (TESTED) = the number of MT-SNVs from the region considered in the gene-based test; MT = mitochondrion.

	HARD			SOFT		
Set	*p*	*n* (ALL)	*n* (TESTED)	*p*	*n* (ALL)	*n* (TESTED)
*CO2*	0.04	5	5	0.03	5	5
*CYB*	0.05	20	20	0.02	20	20
*ND3*	0.10	8	8	<0.01	8	8
*ND4L*	0.19	2	2	0.30	2	2
*ND5*	0.10	35	33	0.14	35	33
*rRNA^12S^*	0.08	11	11	0.05	11	11
*rRNA^16S^*	0.17	13	13	0.07	13	13
MT	0.07	243	226	0.07	243	226

**Table 5:** Haplogroup assignment in HARD and SOFT CAD cases vs. controls prior to further assigning individuals to one of the major European haplogroups. * Indicates that a haplogroup survived multiple testing correction (at FDR < 5%).

	HARD				SOFT			
Haplogroup	OR	Cases (%)	Controls (%)	*p*	OR	Cases (%)	Controls (%)	*p*
G1a	4.86	0.02	<0.01	7.02 × 10^−3^	4.54	0.02	<0.01	1.51 × 10^−3^
G2b1a2	1.73	0.28	0.16	1.20 × 10^−4^ *	1.60	0.26	0.16	2.80 × 10^−5^ *
L2c	0.18	0.01	0.07	2.00 × 10^−4^ *	0.60	0.04	0.07	0.04
M27b	3.99	0.03	0.01	3.79 × 10^−3^	3.00	0.02	0.01	7.42 × 10^−3^
M3a	1.71	0.22	0.13	8.80 × 10^−4^	1.67	0.21	0.13	5.70 × 10^−5^ *
M45a	1.52	0.59	0.39	1.00 × 10^−5^ *	1.42	0.55	0.39	4.00 × 10^−6^ *
M57b1	33.06	0.02	<0.01	1.40 × 10^−4^ *	19.31	0.01	<0.01	9.97 × 10^−4^
M65a1	2.24	0.08	0.04	2.84 × 10^−3^	2.00	0.07	0.04	1.37 × 10^−3^
U2b2	2.56	0.11	0.04	1.20 × 10^−4^ *	2.31	0.09	0.04	2.90 × 10^−5^ *

## Data Availability

The data supporting results reported in this study will be returned to the UK Biobank and available for download to registered researchers on approved applications (https://biobank.ndph.ox.ac.uk/ukb/ukb/docs/ukblink_instruct.html, accessed on 22 February 2022).

## Supplementary Materials

The following supporting information can be downloaded at: https://www.mdpi.com/article/10.3390/genes13030516/s1, Table S1. A complete list of HARD and SOFT CAD case definition codes. Table S2. A complete list of 111 common and low-frequency MT-SNV associations with HARD CAD phenotype. Table S3. A complete list of 111 common and low-frequency MT-SNV associations with SOFT CAD phenotype. Table S4. Haplogroup assignment in HARD CAD cases vs. controls prior to excluding samples with quality scores for haplogroup assignment <0.8 and further assigned individuals to one of the major European haplogroups. Table S5. Haplogroup assignment in SOFT CAD cases vs. controls prior to excluding samples with quality scores for haplogroup assignment <0.8 and further assigned individuals to one of the major European haplogroups.
